# Simultaneous Separation of Eight Lignans in *Forsythia suspensa* by β-Cyclodextrin-Modified Capillary Zone Electrophoresis

**DOI:** 10.3390/molecules23030514

**Published:** 2018-02-26

**Authors:** Jun Liang, Feng-Qiu Gong, Hui-Min Sun

**Affiliations:** Key Laboratory of Chinese Materia Medica (Heilongjiang University of Chinese Medicine), Ministry of Education, Harbin 150040, China; fengqiugong@163.com (F.-Q.G.); hhuiminsun@163.com (H.-M.S.)

**Keywords:** capillary zone electrophoresis, *Forsythia suspensa*, lignans, β-CD

## Abstract

The aim of the study was to develop an alternative capillary zone electrophoresis (CZE) for simultaneous determination of phillyrin (**1**), phillygenin (**2**), epipinoresinol-4-*O*-β-glucoside (**3**), pinoresinol-4-*O*-β-glucoside (**4**), lariciresinol (**5**), pinoresinol (**6**), isolariciresinol (**7**) and vladinol D (**8**) in *Forsythia suspensa*. The structural types of lignans **1**–**8** could be attributed to bisepoxylignans (**1**–**4** and **6**), monoepoxylignans (**5** and **8**) and cyclolignan (**7**). The major difficulties in the CZE separation of **1**–**8** could be summarization as the simultaneous presence of free lignans (**1**, **2** and **5**–**8**) and lignan glucosides (**3** and **4**) and simultaneous occurrence of two pairs of isomers (**3** and **4** as well as **5** and **7**). Without the addition of β-CD and methanol, the resolution of these analytes was quite poor. However, in this study, compounds **1**–**8** were excellently separated from each other within 15 min under optimized conditions with a borax running buffer (40 mM borax, pH 10.30) containing 2 mM β-CD and 5% methanol (*v*/*v*) at the voltage of 20 kV, temperature of 35 °C and detection wavelength of 234 nm. Validation of the method included tests of linearity, precision, repeatability, stability and accuracy. In addition, the method offered inherent advantages such as lower analytical cost, no need of specific columns and use of small amounts of organic solvents and reagents. Finally, this green and economic CZE was successfully applied for the determination of these bioactive components **1**–**8** in *F. suspensa* fruits and its commercial extracts.

## 1. Introduction

*Forsythia suspensa* (Oleaceae) is a perennial herb, which is widely distributed in China, Korea, Japan and many European nations [1]. The fruits of *F. suspensa* have been used as a traditional Chinese medicine for centuries, named ‘Lianqiao’ in Chinese, which has long been used to treat gonorrhea, erysipedas, inflammation, pharyngitis, pyrexia, tonsillitis, ulcers and other diseases. In recent years, reports about the benefits of *F. suspensa* lignans have dramatically increased [1,2]. The ‘Lianqiao’ could be classified into ‘Qingqiao’ and ‘Laoqiao’ according to the maturity levels of the fruits of *F. suspensa*.

Therefore, the determination of the lignans in fruits of *F. suspensa* is very necessary and several analytical methods have been used in recent years. Examples are: thin-layer chromatography (TLC) [3], high-performance liquid chromatographic (HPLC) [4,5] and liquid chromatographic-tandem mass spectrometric (LC-MS) [6] techniques. However, some methods suffered from low resolutions (TLC) and large consumption of organic solvents (HPLC and HPLC-MS). Therefore, it is essential to develop a simple and sensitive analytical method for detecting and separating the lignans in fruits of *F. suspensa.*

Capillary electrophoresis (CE) is an attractive phytochemical analysis tool due to minimum requirements for sample preparation, small sample size, low reagent consumption, high separation efficiency/selectivity, and fast analysis time [7,8,9]. The micellar electrokinetic chromatography (MEKC) methods have been proposed for the analysis of diarylbutyrolactone-type, tetrahydrofuran-type, diarylbutane-type, aryltetralin-type and dibenzocyclooctene-type lignans in *Phyllanthus urinaria*, *P. niruri* and *Schisandra chinensis* [10,11]. Furthermore, non-aqueous and pressurized CE methods have been reported for analysis of dibenzocyclooctene-type lignans in *S. chinensis* for quality evaluation [12,13]. It is well-known that bisepoxylignans are the dominant components in *F. suspensa*. Until now, the analysis of *F. suspensa* lignans using CE has not been reported.

**Figure 1:**
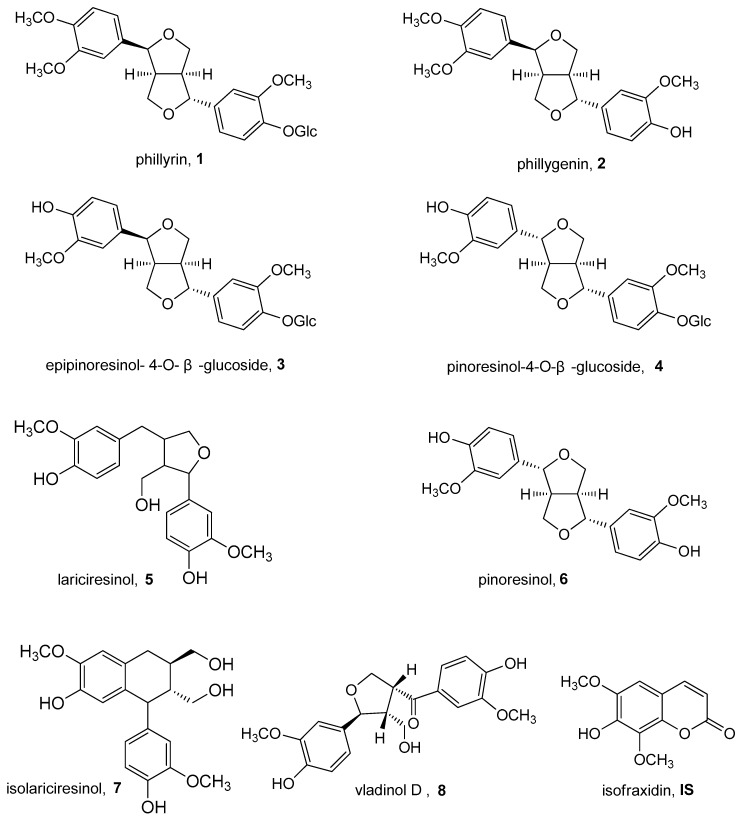
Chemical structures of lignans **1**–**8** and internal standard (IS).

As mentioned above, considering the structural similarity of the investigated analytes in the present work, a β-cyclodextrin-modified capillary zone electrophoresis (β-CD-CZE) with direct UV detection was developed for the simultaneous assay of eight bioactive components **1**–**8** (Figure 1) in *F. suspensa* and its commercial extracts for the first time and gave good results.

## 2. Results and Discussion

### 2.1. Development of Separation Method

In this study, compounds **1**–**8** were determined as phillyrin, phillygenin, epipinoresinol-4-*O*-β-glucoside, pinoresinol-4-*O*-β-glucoside, lariciresinol, pinoresinol, isolariciresinol and vladinol D, respectively. In previous reports [10], MEKC was confirmed to be useful in the analysis of lignans in different plant matrices. Thus, our preliminary electrophoresis experiments were conducted to separate compounds **1**–**8** using MEKC methods. The structural types of compounds **1**–**8** (Figure 1) could be attributed to bisepoxylignans (**1**–**4** and **6**), monoepoxylignans (**5** and **8**) and cyclolignan (**7**). Thus, the major difficulties in the CZE separation of compounds **1**–**8** could be summarized as the simultaneous presence of free lignans (**1**, **2** and **5**–**8**) and lignan glucosides (**3** and **4**) and simultaneous occurrence of two pairs of isomers (**3** and **4** as well as **5** and **7**).

No matter which solvent described in the literature was used as a background electrolyte [10,11], it did not improve the poor separation of compounds **1**–**8**. This fact could be explained by the low-acid property and at least one hydroxy group in each core skeleton of compounds **1**–**8**. In contrast, only methoxy and/or methylenedioxy groups occur in lignans tested in MEKC [10,11]. Thus, CZE was further attempted in this study. After many attempts, a β-CD modified CZE was finally implemented and optimized based on borax running buffer in alkali. Method development [14] was achieved by mainly optimizing pH, borax concentrations of the buffer, β-CD and methanol for analysis.

#### 2.1.1. Effect of pH

**Figure 2:**
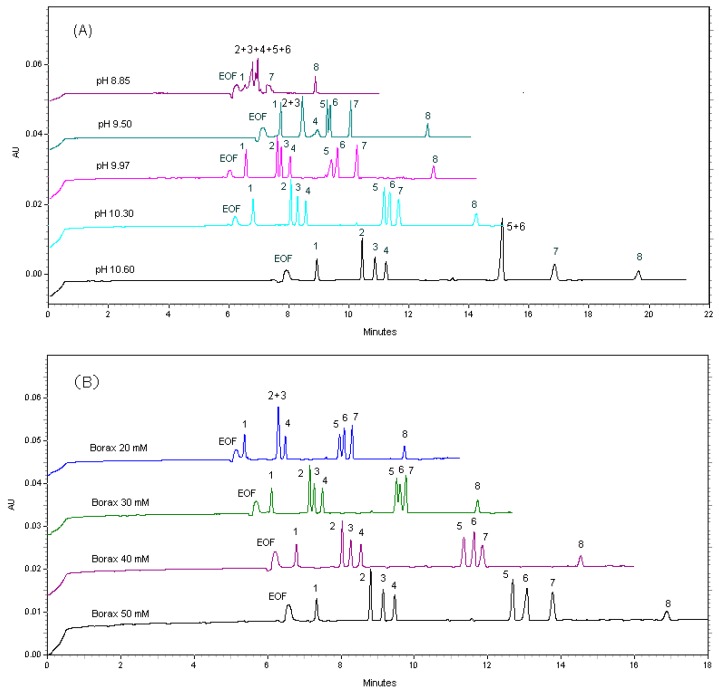
Effects of pH and buffer concentration on the migration time and separation of analytes. (**A**) 40 mM borax with 2 mM β-CD and 5% (*v*/*v*) methanol at different pH vaules; (**B**) different concentrations of borax with 2 mM β-CD and 5% (*v*/*v*) methanol at pH 10.30.

The pH of the buffer has been confirmed to be a very important parameter here because only compound **1** is not charged, while compounds **2**–**4** and **5**–**8** could be charged −1 and −2 at high pH values, respectively. Therefore, to investigate the effect of pH on migration behaviors of compounds **1**–**8**, five different pH values, varying from 8.85 to 10.60 (8.85, 9.50, 9.97, 10.30 and 10.60), were used based on 40 mM borax with 2 mM β-CD and 5% (*v*/*v*) methanol. The results showed a gradual increase of migration time with increasing pH values from 8.85 to 10.60 for all of the analytes **1**–**8** (Figure 2A).

We observed that, if the pH values were lower than 9.97, the total run time was shorter within 13 min, while the resolution for the compounds **2**–**6** was worse. When high pH 10.60 was applied, the resolution between peaks **2**–**4** was obviously better, whereas the resolution between peaks **5** and **6** was quite poor and co-eluted. Furthermore, the total analysis time was much longer and has moved up to 20 min. However, a compromise was obtained at pH 10.30, and all compounds **1**–**8** could be separated baseline in short time within 15 min. Therefore, pH 10.30 was selected for the further experiments. It is well-known that electroosmotic flow (EOF) generally increases with an increase in the pH of back-ground electolyte. However, it was noted here that the order of the EOF magnitude (Figure 2A) seems to be different from the general order. This may be explained by the fact that the damaged capillary column was replaced after observation of the influences at pH 9.50.

#### 2.1.2. Effect of Borax Concentrations

Borax will form a covalent complex with each compound tested. Figure 2B showed the effect of borax concentrations on the separation of the compounds **1**–**8**. Different concentrations of borax buffer ranging from 20 to 50 mM with 2 mM β-CD and 5% (*v*/*v*) methanol at pH 10.30 were used at 20 kV as applied voltage and 35 °C, to study the effects of buffer concentrations on the migration time of the lignans **1**–**8**. It was found that compounds **2** and **3** as well as **5** and **6** were not well separated at all using 20 and 30 mM of borax buffer, respectively. The optimum borax concentration ensuring the separation of all compounds down to the baseline was found to be 40 and 50 mM. The 50 mM borax buffer did increase the resolutions of compounds **6** and **7**, whereas too long migration time and poor peak symmetry of compound **8** was observed. Thus, 40 mM borax buffer was chosen for further experiments. In this study, it was also noted that the migration time of EOF was increased by increasing borax concentrations from 20 to 50 mM.

#### 2.1.3. Effect of β-CD

**Figure 3:**
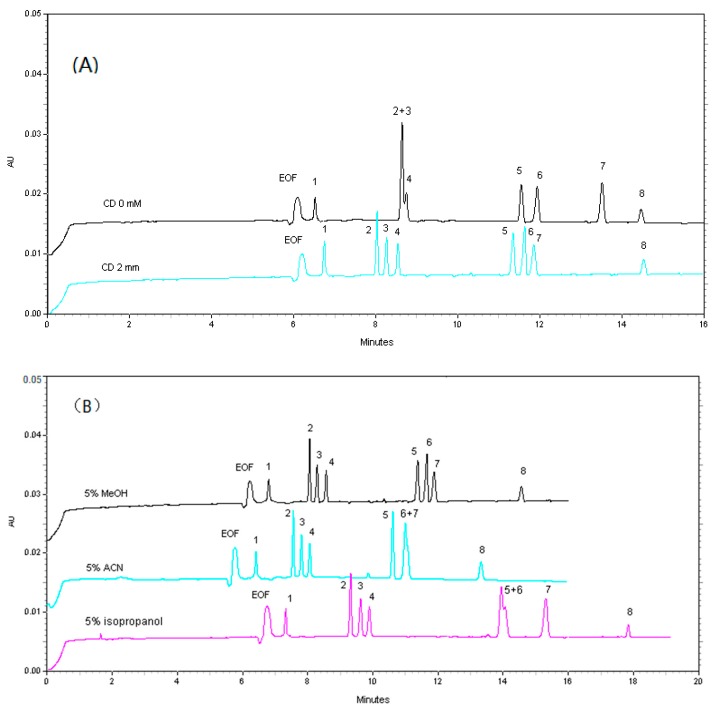
Effects of β-CD and organic modifiers on the migration time and separation of analytes. (**A**) 40 mM borax with different concentrations of β-CD and 5% (*v*/*v*) methanol at pH 10.30; (**B**) 40 mM borax with 2 mM β-CD and different organic modifiers at pH 10.30. Labels of compounds are the same as for Figure 2.

The β-CD modified CZE method has been widely applied in enantioseparations [15]. In this study, there are two pairs of isomers (**3** and **4** as well as **5** and **7**) which can interact with β-CD. Thus, the effects of the addition of β-CD on separation efficiency were investigated in 40 mM borax with 5% (*v*/*v*) methanol at pH 10.30. It was also noted that the mobility of compounds **2**–**4** were obviously different before and after the addition of β-CD. As shown in Figure 3A, a 2 mM β-CD could result in the baseline separation of peaks **1**–**8**, so this concentration was chosen as the optimal condition in subsequent experiments.

#### 2.1.4. Effect of Organic Modifiers

The use of organic solvents such as MeOH, ACN and isopropanol in the buffer could improve selectivity, and thus would expand the migration window [15]. According to factors mentioned above, the resolution was obtained with an electrolyte containing 40 mM borax with 2 mM β-CD at 5% MeOH, 5% ACN or 5% isopropanol at pH 10.30 (Figure 3B). When 5% ACN and 5% isopropanol were used, compounds **6** and **7** as well as **5** and **6** could not be separated at all, respectively. Considering resolutions, analysis time and stability of migration time, 5% MeOH was the first selection.

Based on all the factors described above, an optimal CZE condition, which was 40 mM with 2 mM β-CD and 5% MeOH at pH 10.30, was established for the simultaneous separation of phillyrin (**1**), phillygenin (**2**), epipinoresinol-4-*O*-β-glucoside (**3**), pinoresinol-4-*O*-β-glucoside (**4**), lariciresinol (**5**), pinoresinol (**6**), isolariciresinol (**7**) and vladinol D (**8**) in *F. suspensa*.

### 2.2. Method Validation

To apply the method to real samples and insure the accuracy of the method, we chose isofraxidin as the internal standard (IS) compound. The linear regression between the ratios of peak areas of analytes to that of IS and the corresponding concentrations analysis were performed under the optimum conditions.

As shown in Table 1, good linearity was seen in the certain concentration range with the coefficient of determination (*R^2^*) higher than 0.99 for all the analytes **1**–**8**. The limit of detections (LODs) and the limit of quantifications (LOQs) for all standard analytes were shown in Table 1. The RSDs of migration time and peak areas (Table S1) were less than 1% and 2% for intra-day (*n* = 6) as well as less than 1% and 5% for inter-day (*n* = 3), respectively. This showed good precision for the whole method.

### 2.3. Application and Recovery

**Figure 4:**
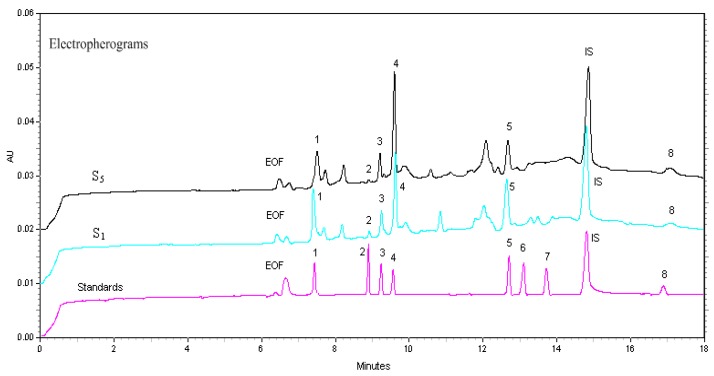
Electropherograms of *F. suspensa* extract (S_1_), *F. suspensa* fruits (S_5_) and reference standards. Labels of compounds are the same as for Figure2, IS: internal standard.

A number of biological activities of these compounds were previously reported [1,2]. The phillyrin, phillygenin, pinoresinol-4-*O*-β-glucoside were selected to be quantitative constituents in *F. suspensa* extract in the 2010 and 2015 editions of Chinese Pharmacopoeia. Therefore, qualitative and quantitative analysis of these characteristic constituents could play an essential role in evaluating and controlling the quality of *F. suspensa.* Here, an alternative β-CD-CZE method was readily established for determination of the bioactive compounds **1**–**8** in *F. suspensa*. Representative CZE electropherograms of reference standards as well as samples S_1_ and S_5_ are shown in Figure 4.

The samples S_1_ and S_5_ are typical commercial extracts and raw materials of *F. suspensa*, respectively. The peaks were identified by comparison of its UV spectra and the migration times with those of reference standards, and by spiking the sample solution with standards. The content of lignans **6** and **7** was too low to qualify. The amounts of compounds **1**, **2**, **3**, **4**, **5** and **8** determined in the analyzed samples have been described in Table 2. The results showed that there were remarkable differences in the contents of the lignans detected in four batches of *F. suspensa* raw materials and four batches of commercial *F. suspensa* extracts. These variations may explained by geographical source, cultivation, harvest, storage, and processing of the herb [4,5,6].

The recoveries were performed by adding a certain amount of mixture standards (about 50%, 100% and 150% of the content) to real samples. The recoveries ranged from 94% to 104% for all the samples with RSD within 4%. The above results displayed the good reliability and accuracy for the measurement of these constituents. The recovery was calculated as follows: recovery (%) = 100**%**× (amount found-original amount)/amount spiked, as shown in Table S2. Compared with other analytical methods, the developed β-CD-CZE possessed good linear ranges and good sensitivity. For instance, the LOQ was 3.28 μg/mL for phillyrin (**1**), which was 1.5 times lower than those from the HPLC-UV detection method [5]. Furthermore, this method showed inherent advantages such as no need for specific columns, organic solvents and reagents.

## 3. Materials and Methods

### 3.1. Chemicals and Regents

HPLC-grade methanol was purchased from Merck (Darmstadt, Germany). Sodium tetraborate (Na_2_B_4_O_7_·10H_2_O), disodium hydrogen phosphate dodecahydrate (Na_2_HPO_4_·12H_2_O) and sodium hydroxide (NaOH) were analytical grade and made in Tianjin, China. Throughout the study, deionized water was prepared by a Milli-Q water system (Millipore, Bedford, MA, USA). All other chemicals were of analytical grade. Reference standards of lignans **1**–**8** were isolated from the fruits of *F. suspensa* in our laboratory whose structures were identified on the basis of spectroscopic analysis [5,16,17]. The purity of each compound was determined to be > 98% by normalization of the peak areas detected by HPLC analysis.

### 3.2. Electrophoretic Procedures

Separations of lignan standards and *F. suspensa* samples were carried out with a P/ACE MDQ capillary electrophoresis instrument (Beckman Coulter, Fullerton, CA, USA). An integrated P/ACE 32 Karat Station (software version 4.0) was used to perform the data collection and to control the operational variables of the system. Separation was carried out in an unmodified fused silica capillary (48.5 cm × 50 μmi.d., effective length 40 cm) (Yongnian Optical Fiber Factory, Handan, Hebei, China). Both the capillary and samples were thermostatted to 35 °C. The samples were injected with a pressure of 0.5 psi for 5 s. The separation voltage was raised linearly within 0.5 min from 0 to 20 kV. Detection was done with direct UV (Beckman Coulter, Fullerton, CA, USA) monitoring using a photodiode array detector at wavelength 234 nm.

A new capillary from Yongnian Optical Fiber Factory (Handan, Hebei, China) was activated by washing consecutively with each of 0.1 M phosphoric acid (15 min), water (10 min), 0.1 M sodium hydroxide (15 min), and water (10 min). At the beginning of each working day, the capillary was prewashed with 0.1 M phosphoric acid (2 min), water (2 min), 0.1 M NaOH for 2 min, water (2 min) and running buffer (2 min), respectively. Between different analyses, the capillaries were consecutively rinsed with 0.1 M NaOH (1 min), water (1 min) and running buffer (1 min).

### 3.3. Standard Solutions Preparations

For the method developments, standard stock solutions of 1.0 mg/mL were prepared in methanol, and then diluted with methanol to the appropriate concentration to establish calibration curves. The stock solutions were stored in a refrigerator (+4 °C).

### 3.4. Sample Preparations

The dried powder of *F. suspensa* fruits (100 mesh, 1g) in a 5 mL volumetric flask were extracted with 20% methanol in ultrasonic bath (pulse energy 70 kHz) for 30 min × 3 times. The total volume of extract was adjusted to 15 mL with 20% methanol. The mixture was centrifuged at 4000 rpm and 4 °C for 5 min. The supernatant was filtered by a 0.45 um pore size filter and then used as sample solution. Before injection, 100 uL of isofraxidin (200 ug/mL) used as internal standard was added to 900 uL sample solution. All samples were determined in triplicate. 

For *F. suspensa* extract analysis, 50 mg extract was dissolved in 5 mL 20% methanol, the solution was filtered by 0.45 um pore size filter, then 100 uL of isofraxidin (200 ug/mL) used as internal standard was added to 900 uL sample solution before injection.

## 4. Conclusions

This study aims to design a CZE separation as a green, simple and economic method for simultaneous determination of multiple lignans in *F. suspensa*. The structural types of these lignans **1**–**8** could be attributed to bisepoxylignans (**1**–**4** and **6**), monoepoxylignans (**5** and **8**) and cyclolignan (**7**). These compounds were also characterized by simultaneous presence of free lignans (**1**, **2** and **5**–**8**) and lignanglucosides (**3** and **4**) and by simultaneous occurrence of two pairs of isomers (**3** and **4** as well as **5** and **7**). This study represents the first separation of phillyrin (**1**), phillygenin (**2**), epipinoresinol-4-*O*-β-glucoside (**3**), pinoresinol-4-*O*-β-glucoside (**4**), lariciresinol (**5**), pinoresinol (**6**), isolariciresinol (**7**), and vladinol D (**8**) using a capillary electrophoresis method. Compared with other analytical methods, this β-CD modified CZE method offered inherent advantages such as lower analytical cost, no need for specific columns and use of small amounts of organic solvents and reagents.

## Figures and Tables

**Table 1:** Linearity of CZE (Capillary Zone Electrophoresis) method of different analytes **1**–**8** ^a^.

No. ^b^	Regression Equation ^c^	Linear Range (μg/mL)	LOD (μg/mL)	LOQ (μg/mL)	^d^ *R*^2^
**1**	*y* = 0.0094*x* − 0.117	3.28–131.25	0.94	3.28	0.9978
**2**	*y* = 0.0124*x* − 0.0963	3.75–150.00	1.07	3.75	0.9962
**3**	*y* = 0.0094*x* − 0.1055	4.19–167.50	1.20	4.19	0.9965
**4**	*y* = 0.0092*x* − 0.0868	3.69–147.50	1.05	3.69	0.9919
**5**	*y* = 0.0185*x* − 0.1889	3.50–140.00	1.00	3.50	0.9944
**6**	*y* = 0.0199*x* − 0.3224	4.38–175.00	1.25	4.38	0.9963
**7**	*y* = 0.0134*x* − 0.1248	3.72–148.75	1.06	3.72	0.9945
**8**	*y* = 0.0091*x* − 0.1021	3.00–120.00	0.86	3.00	0.9953

^a^ Running buffer: 40 mM sodium borate with 2 mM β-CD and 5% (*v*/*v*) methanol at pH 10.30; ^b^ Quantitated with a calibration curve at = 234 nm; ^c^ Area ratio equation, where *y* was the area ration between analytes and internal standard and *x* was the concentration (μg/mL) of analytes; ^d^ *R*^2^= correlation coefficient, *n* = 6.

**Table 2:** Contents of compounds **1**–**8** in different *F. suspensa* extracts and *F. suspensa* fruits*.*

No.	Types	Contents (μg/g)					
		1	2	3	4	5	8
S_1_	CE ^a^	3766 ± 135	856 ± 20	2618 ± 73	6393 ± 103	3528 ± 139	1894 ± 75
S_2_	CE ^a^	3942 ± 151	1693 ± 34	4039 ± 106	8972 ± 187	6603 ± 103	2904 ± 75
S_3_	CE ^a^	36,887 ± 790	4707 ± 109	26,681 ± 344	70,652 ± 978	47,686 ± 935	20,869 ± 326
S_4_	CE ^a^	35,529 ± 800	8825 ± 232	28,492 ± 721	71,299 ± 1023	67,462 ± 1013	18,304 ± 289
S_5_	Fruits ^b^	2009 ± 70	506 ± 15	1719 ± 42	4942 ± 74	1594 ± 78	1425 ± 58
S_6_	Fruits ^b^	1137 ± 20	517 ± 14	779 ± 11	1783 ± 54	761 ± 14	1104 ± 25
S_7_	Fruits ^b^	1110 ± 24	606 ± 17	1022 ± 27	1985 ± 89	790 ± 29	3497 ± 167
S_8_	Fruits ^b^	-	983 ± 24	-	611 ± 10	491 ± 12	793 ± 15

^a^ Commercial extracts of *F. suspensa* fruits. ^b^ *F. suspensa* raw materials.

## Supplementary Materials

The following are available online, Table S1: Intra- and inter-day variability for the assay of the 8 constituents, Table S2: Recoveries of the 6 constituents by use of the established CZE method.
