# Supplementary material for: Simultaneous Separation of Eight Lignans in Forsythia suspensa by β-Cyclodextrin-Modified Capillary Zone Electrophoresis
Source: Molecules. 2018 Feb 26;23(3):514. doi: 10.3390/molecules23030514 (PMC6017137; doi:10.3390/molecules23030514)
Supplement: Supplementary file 1 [file molecules-23-00514-s001.pdf]

Table S1

Intra- and inter-day variability for the assay of the 8 constituents[a]

Compounds	Intra-day(n=6)				Inter-day(n=3)			
	t_R (mean ± SD)	RSD(%)[a]	Area (mean ± SD)	RSD(%)[a]	t_R (mean ± SD)	RSD(%)[a]	Area (mean ± SD)	RSD(%)[b]
1	7.358 ± 0.043	0.58	12929 ± 221	1.71	7.338 ± 0.064	0.87	12879 ± 355	2.76
2	8.871 ± 0.055	0.62	30994 ± 456	1.47	8.876 ± 0.060	0.68	29802 ± 1380	4.63
3	9.213 ± 0.045	0.49	23464 ± 209	0.89	9.192 ± 0.073	0.79	20546 ± 723	3.52
4	9.537 ± 0.091	0.95	19488 ± 265	1.36	9.542 ± 0.094	0.99	18528 ± 406	2.19
5	12.713 ± 0.095	0.75	33717 ± 502	1.49	12.658 ± 0.116	0.92	33829 ± 1370	4.05
6	13.079 ± 0.069	0.53	34551 ± 321	0.93	13.158 ± 0.062	0.47	36781 ± 1365	3.71
7	13.725 ± 0.066	0.48	30599 ± 539	1.76	13.771 ± 0.116	0.84	35232 ± 715	2.03
8	16.863 ± 0.096	0.57	11504 ± 159	1.38	16.842 ± 0.099	0.59	11628 ± 355	3.05

[a] Analyte concentrations were 30 μg/mL

[b] RSD (%) = (SD/mean) ×100.

Table S2.

Recoveries of the 6 constituents by use of the established CZE method

Compound	Amount spiked (μg/mL)	Amount found-original amount (μg/mL)[a]	Recovery(%)[b]	RSD (%)
**1**	26.83	25.68	95.74±1.81	1.89
	33.54	34.17	101.91±3.31	3.25
	40.25	39.36	97.85±2.50	2.56
**2**	6.76	6.38	94.46±2.43	2.57
	8.45	8.55	101.23±1.60	1.58
	10.14	9.69	95.61±1.33	1.39
**3**	22.97	22.64	98.69±3.01	3.05
	28.71	27.96	97.47±1.64	1.68
	34.45	34.1	99.05±2.47	2.49
**4**	66.02	66.78	101.27±3.64	3.59
	82.53	81.39	98.64±2.93	2.97
	99.04	98.57	99.57±1.89	1.90
**5**	21.30	22.06	103.63±3.24	3.13
	26.62	25.86	97.15±1.44	1.48
	31.94	32.31	101.22±1.59	1.57
**8**	19.05	17.98	94.48±1.76	1.86
	23.81	23.05	96.89±2.30	2.37
	28.57	29.2	102.26±2.54	2.48

[a] Calculated by amount found-original amount. Data were means of three experiments.

[b] Calculated as recovery (%) = 100% ×(amount found-original amount)/amount spiked
